# Learning from B_12_ enzymes: biomimetic and bioinspired catalysts for eco-friendly organic synthesis

**DOI:** 10.3762/bjoc.14.232

**Published:** 2018-10-02

**Authors:** Keishiro Tahara, Ling Pan, Toshikazu Ono, Yoshio Hisaeda

**Affiliations:** 1 Department of Material Science, Graduate School of Material Science, University of Hyogo, 3-2-1, Kouto, Kamigori, Ako 678-1297, Japan; 2 Department of Chemistry, Northeast Normal University, Changchun 130024, P. R. China; 3 Department of Chemistry and Biochemistry, Graduate School of Engineering, Kyushu University, 744 Motooka, Nishi-ku, Fukuoka 819-0395, Japan; 4 Center for Molecular Systems (CMS), Kyushu University, Fukuoka 819-0395, Japan; 5 PRESTO, Japan Science and Technology Agency (JST), 4-1-8 Honcho, Kawaguchi, Saitama 332-0012, Japan

**Keywords:** dehalogenation, electrolysis, green chemistry, heptamethyl cobyrinate, methyl transfer, 1,2-migration, photosensitizer, vitamin B_12_

## Abstract

Cobalamins (B_12_) play various important roles in vivo. Most B_12_-dependent enzymes are divided into three main subfamilies: adenosylcobalamin-dependent isomerases, methylcobalamin-dependent methyltransferases, and dehalogenases. Mimicking these B_12_ enzyme functions under non-enzymatic conditions offers good understanding of their elaborate reaction mechanisms. Furthermore, bio-inspiration offers a new approach to catalytic design for green and eco-friendly molecular transformations. As part of a study based on vitamin B_12_ derivatives including heptamethyl cobyrinate perchlorate, we describe biomimetic and bioinspired catalytic reactions with B_12_ enzyme functions. The reactions are classified according to the corresponding three B_12_ enzyme subfamilies, with a focus on our recent development on electrochemical and photochemical catalytic systems. Other important reactions are also described, with a focus on radical-involved reactions in terms of organic synthesis.

## Review

### 1. Introduction

#### 1-1. Redox and coordination chemistry of B_12_

**Figure 1:**
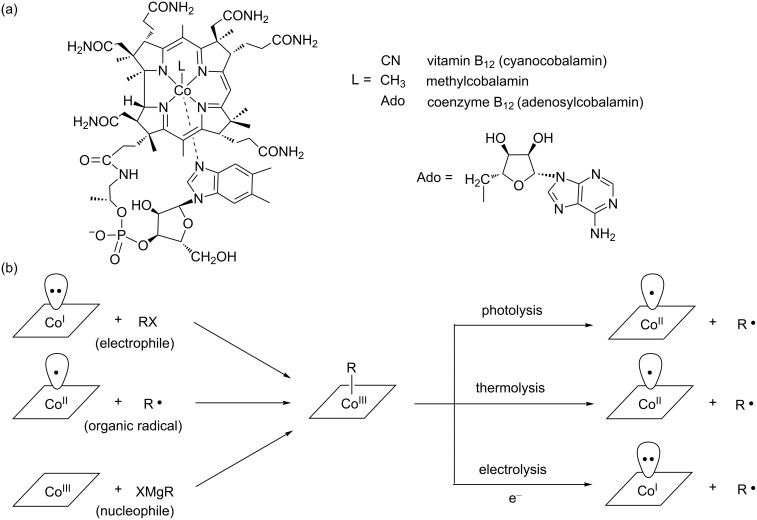
(a) Structure and (b) reactivity of B_12_.

Cobalamins (B_12_) are naturally occurring cobalt complexes with unique structures that play various important roles in vivo [1–5]. In B_12_, the cobalt center is coordinated by four equatorial pyrroles of the corrin ring and 2,3-dimethylbenzimidazole as a lower axial ligand (Figure 1a) [6–8]. The cobalamin with an upper ligand is termed vitamin B_12_ (a cyanide group), methylcobalamin (a methyl group), and adenosylcobalamin (an adenosyl group), respectively. The oxidation state of cobalt ions in B_12_ ranges from +1 to +3. Each oxidation state of cobalamins exhibits quite different ligand-accepting abilities and reactivities. Cob(III)alamins strongly favor 6-coordination with 2,3-dimethylbenzimidazole in homogeneous solutions at physiological pH (denoted as base-on form). In particular, cob(III)alamins with upper alkyl ligands are quite interesting because of their structural relevance to methylcobalamin and adenosylcobalamin (coenzyme B_12_) that serve as organometallic cofactors in B_12_-dependent enzymes. The photolysis (thermolysis) of alkylcob(III)alamins leads to the formation of the corresponding alkyl radical and cob(II)alamin with homolytic Co(III)–C bond cleavage (Figure 1b). This high lability is attributed to a relatively weak Co(III)–C bond, as exemplified by its bond dissociation energies of 30 kcal/mol in coenzyme B_12_ and 37 kcal/mol in methylcobalamin in base-on forms [9]. Cob(II)alamin favors 5-coordination in the homogeneous solutions at physiological pH [10]. It is paramagnetic and has an unpaired electron in the axial d*z*^2^ orbital. It acts as a high efficient “radical trap” and reacts with alkyl radicals to yield alkylcob(III)alamin (Figure 1b). Four-coordinated cob(I)alamin has a paired electron in the axial d*z*^2^ orbital, resulting in high nucleophilicity with a Pearson constant of 14 [11]. It is slightly basic, with a p*K*_a_ lower than 1 for the Co–H complex [12]. The “supernucleophilic” cob(I)alamin is found in many enzymes such as methionine synthetases, adenosyltransferases, and reductive dehalogenases. In addition, the reactivity of cob(I)alamin has been investigated using various electrophiles such as alkyl halides [13], vinyl halides [14–16], aryl halides [17–18] and epoxides [19–20] in homogeneous solutions (Figure 1b).

#### 1-2. Design of biomimetic and bioinspired B_12_ catalytic systems

**Figure 2:**
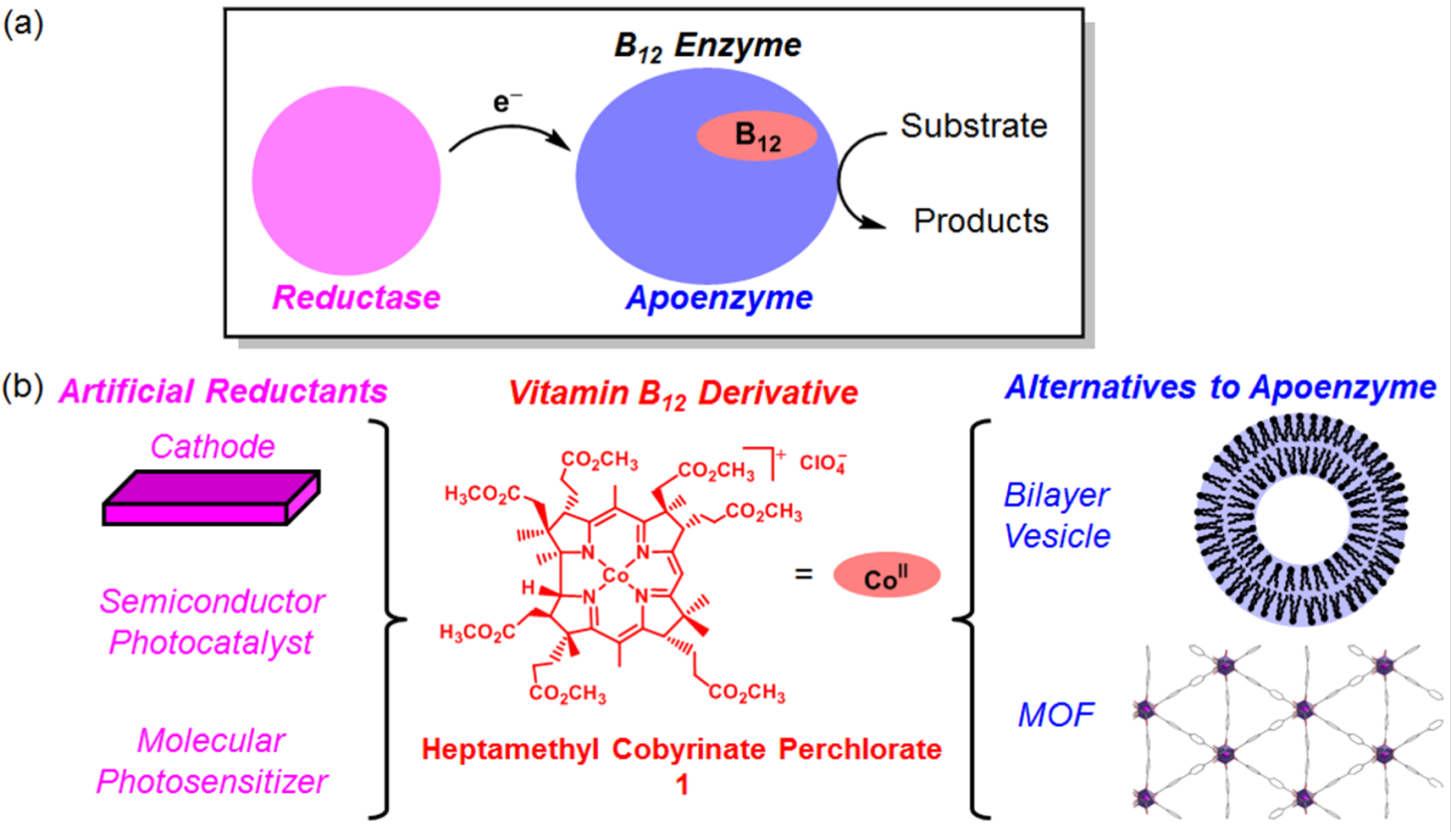
(a) Schematic representation of B_12_ enzyme-involving systems. (b) Construction of biomimetic and bioinspired catalytic systems by combining functional equivalents of B_12_ enzyme-involving systems as components.

Schematic representations of B_12_ enzymes and enzyme-involving systems are shown in Figure 2a. The remarkable in vivo and in vitro characters of B_12_ are summarized as follows:

1. B_12_ shows good accessibility to Co(I) species with a redox potential (the Co(II)/Co(I) couple in the base-off form) of −500 mV vs the standard hydrogen electrode [21], because of the monoanionic corrin ligand.
2. B_12_ is reduced to Co(I) species in the active center by reductases in sustainable processes.
3. The partially π-conjugated system of the corrin ring is less easy to be adducted by free radicals than those of porphyrins.
4. B_12_ is bound to a number of proteins and acts as a module.
5. Different chemical functions of B_12_ are exploited by bound apoenzymes.
6. B_12_ is recycled or reactivated in vivo as observed in methyonine synthetases.

Understanding the mechanisms of B_12_ enzyme reactions and the role of B_12_ is very important from the viewpoint of bioinorganic and organometallic chemistry, organic syntheses, and catalysts. Despite extensive research, reproducing B_12_ enzyme reactions in vitro had been difficult in homogenous solutions.

Construction of sustainable catalytic systems inspired by B_12_ enzymes is another important issue that must be addressed for green chemistry. Due to the above-mentioned unique redox and coordination chemistry, vitamin B_12_ and its derivatives [22] are used as effective homogenous catalysts in various organic reactions [23–25], although an excess of chemical reductants are often used to activate B_12_ to the Co(I) species. Green catalytic systems capable of activating B_12_ have not been reported in the literature, with the exception of electrocatalytic systems [26–27].

To achieve functional simulations of B_12_ enzymes under non-enzymatic conditions, our strategy is to fabricate the artificial enzymes by combining a functional equivalent of B_12_ and that of an apoenzyme (Figure 2b). We have been exploring the utility of hydrophobic B_12_ model complexes, such as heptamethyl cobyrinate perchlorate **1**, that possess ester groups in place of the peripheral seven-amide moieties [28–29]. **1** was developed by Eschenmoser et al. as a model complex for the total synthesis of vitamin B_12_ [30]. Indeed, in the crystal structure, **1** maintained the same corrin framework as natural B_12_ [31]. We combined the hydrophobic B_12_ derivatives with bilayer vesicles [32–33], a protein [34], organic polymers [35–40], and metal organic frameworks (MOFs) [41]. Furthermore, to construct green catalytic systems inspired by B_12_ enzymes, we combined the hydrophobic B_12_ derivatives with a functional equivalent of reductases. In the resultant catalytic systems, the Co(I) species was generated through electron transfers from the cathodes [42–43], semiconductors [44], or molecular photosensitizers [45] to the B_12_. In this review, we summarize the biomimetic and bioinspired catalytic reactions with B_12_ enzyme functions, with a focus on our recent work on electrochemical and photochemical systems.

### 2. 1,2-Migrations of functional groups

**Scheme 1:**
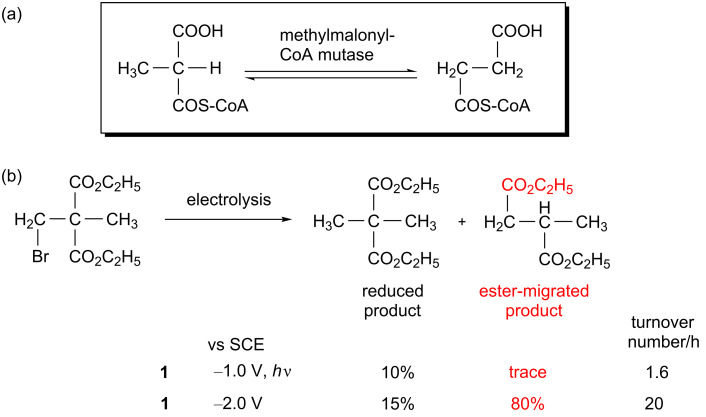
(a) Carbon-skeleton rearrangement mediated by a coenzyme B_12_-depenedent enzyme. (b) Electrochemical carbon-skeleton rearrangement mediated by **1**.

Enzymes using radical species are models of good catalysts for chemists because they efficiently mediate difficult organic reactions under mild conditions [46–51]. In some catalysis mediated by B_12_ enzymes, the high reactivity of the adenosyl radical is exploited for isomerization. The microenvironments provided by the apoenzymes activate and cleave the Co(III)–C bond of the B_12_ coenzyme B_12_ in a homolytic fashion to produce an adenosyl radical [52–53]. In methylmalonyl-CoA mutase (MMCM), the conversion from *R*-methylmalonyl-CoA to succinyl-CoA (Scheme 1a) starts with hydrogen abstraction by the adenosyl radical.

#### 2-1. Electrochemical catalytic reactions

We deeply investigated the electrochemical catalytic reactions mediated by **1** and related complexes and succeeded in the functional simulations of MMCM-type 1,2-migration reactions [42]. For example, when 2,2-bis(ethoxycarbonyl)-1-bromopropane was selected as a model substrate, the 1,2-migration of carboylic ester (80%) and some simple reduction product (20%) were obtained under controlled-potential electrolysis at −2.0 V vs SCE in the presence of catalyst **1** in DMF (Scheme 1b) [54]. There were different ratios for the simple reduced product and the ester-migrated product, depending on the reaction conditions. Mechanistic investigations revealed that the formation of the two-electron-reduced species of Co(III)-monoalkylated complex of **1** was vital for carbon-skeleton rearrangement reactions. It was also discovered that the 1,2-migration of the carboxylic ester group proceeded via an anionic intermediate. To clarify the migratory aptitude of the functional groups, several kinds of substrates with an electron-withdrawing group were utilized. The yields of the migrated products increased in the order of CN < CO_2_R < COR [54]. For alkyl halides with two carboxylic ester groups that differ in their bulkiness, the yields of the migrated products are higher for the smaller ester group [55].

**Scheme 2:**
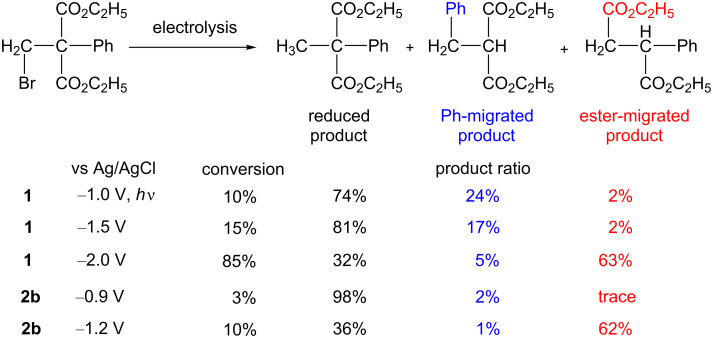
Electrochemical carbon-skeleton arrangements mediated by B_12_ model complexes.

Furthermore, we succeeded in tuning selectivity in the 1,2-migration of a functional group mediated by **1** by controlling the electrolysis potential (Scheme 2) [56]. The electrolysis of diethyl 2-bromomethyl-2-phenylmalonate at −2.0 V vs Ag/AgCl yielded carboxylic ester migrated product as the major product. Conversely, the electrolysis of the substrate at −1.0 V vs Ag/AgCl through light irradiation, as well as at −1.5 V vs Ag/AgCl in the dark, yielded the simple reduced product and the phenyl migrated product. The cathodic reactivity of the monoalkylated complex of **1** was found to be critical to the selectivity of the migrating group.

**Figure 3:**
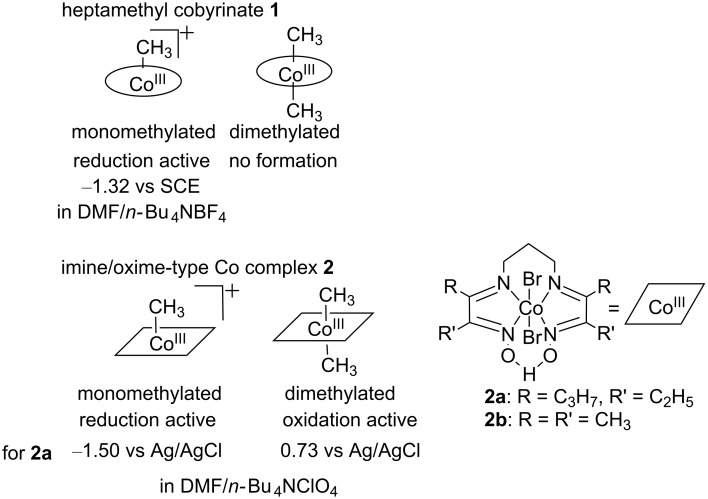
Key electrochemical reactivity of **1** and **2** in methylated forms.

Interestingly, the electrochemical carbon-skeleton rearrangement reactions were successfully mediated by simple B_12_ model complexes **2** (Figure 3). The imine/oxime-type square planar ligands of cobalt complexes **2** are superior to porphyrin ligands in terms of the model for the corrin framework of B_12_; both the imine/oxime-type and corrin ligands are monoanionic [57–60]. The imine/oxime-type cobalt complex **2** can be isolated in both the monoalkylated and dialkylated forms [59–60]. This is in contrast to **1**; **1** cannot be dialkylated because of steric hindrances [42]. The Co(III)-monoalkylated complex can be electrochemically reduced to form Co(I) species and a Co(III)-dialkylated complex through disproportionation. The resulting Co(III)-dialkylated complex shows different electrochemical reactivity. It can be electrochemically oxidized to form the Co(III)-monoalkylated complex. These electrochemical reactivities are exemplified by those of the Co(III)–CH_3_ and Co(III)–(CH_3_)_2_ complexes of compound **2a** in Figure 3. In the electrolysis, the reduction of the Co(III)-monoalkylated complex and the oxidation of the Co(III)-dialkylated complex proceeded at the cathode and anode, respectively [61]. These processes were coupled to achieve the 1,2-migration of functional groups. Further investigations with diethyl 2-bromomethyl-2-phenylmalonate as a substrate confirmed that the carboxylic ester-migrated product was formed via not a radical, but a cationic intermediate that was generated by the fragmentation to the monoalkylated complex at the anode (Scheme 2).

#### 2-2. Artificial enzyme-mediated reactions

**Scheme 3:**
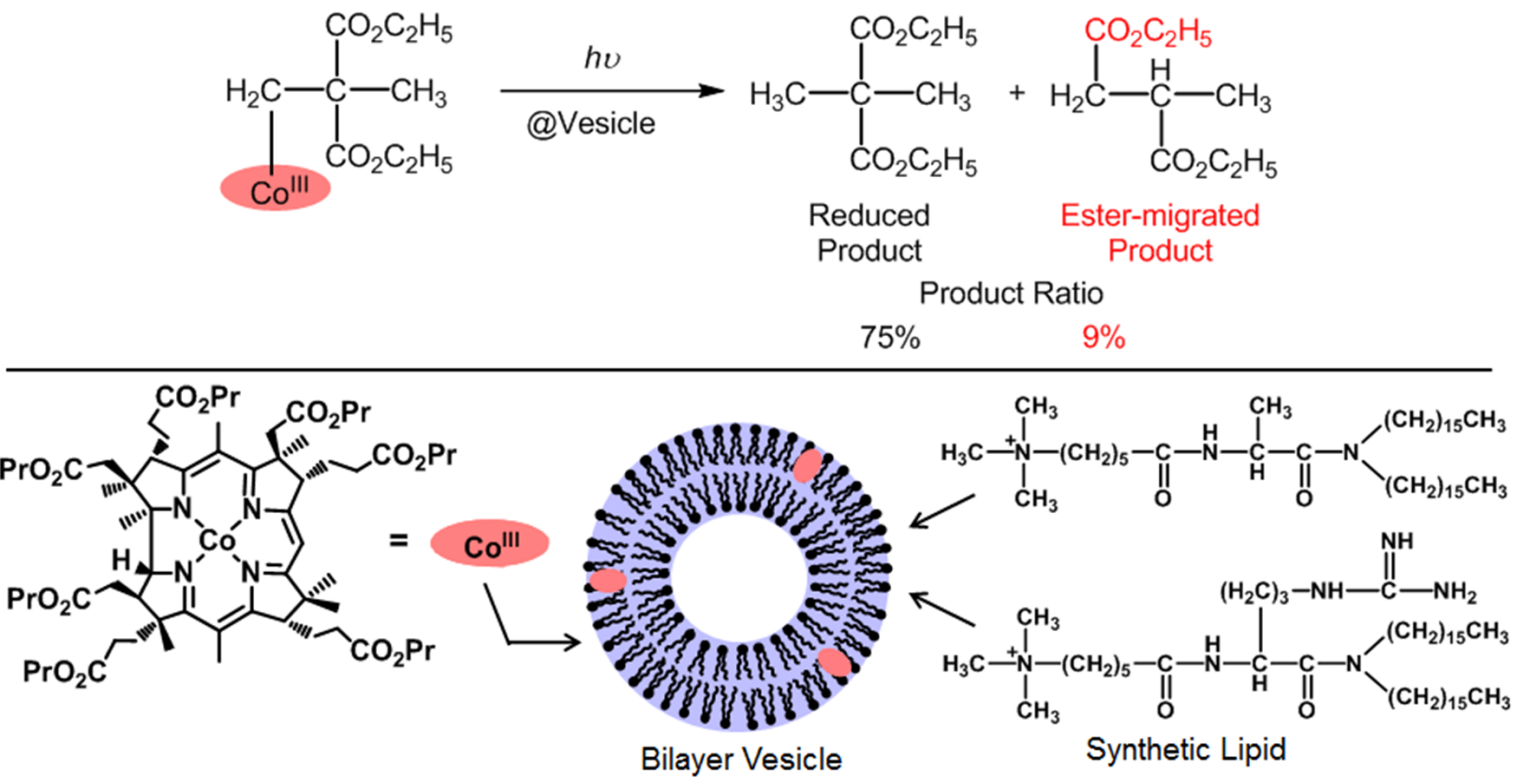
Carbon-skeleton arrangements mediated by B_12_-vesicle artificial enzymes.

A vesicle-type B_12_ artificial enzyme was constructed by combining bilayer vesicles composed of synthetic lipids and alkylated complexes of heptapropyl cobyrinate (Scheme 3) [32–33]. The alkylated B_12_ model complexes were introduced into the vesicle in aqueous solutions through non-covalent hydrophobic interactions and irradiated with a 500 W tungsten lamp to result in the homolytic cleavage of the Co(III)–C bonds. The carbon-skeleton rearrangements were achieved in the vesicle due to cage effects in the apoenzyme model. Conversely, such reactions hardly proceeded in homogenous solutions. The yields of the migration products increased in order of CN ~ CO_2_C_2_H_5_ < COCH_3_. A cyclophane-type B_12_ artificial enzyme also mediated similar carbon-skeleton rearrangements [32].

**Scheme 4:**
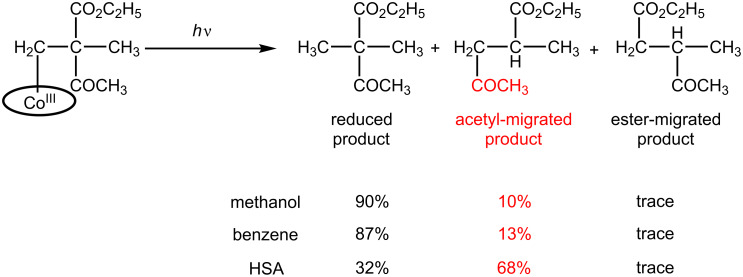
Carbon-skeleton arrangements mediated by B_12_-HSA artificial enzymes.

We developed another artificial enzyme composed of human serum albumin (HSA) and heptapropyl cobyrinate [34]. It is known that HSA acts as a carrier for in vivo hydrophobic molecules. Hydrophobic B_12_ model complexes were successfully incorporated into the HSA. The incorporated amounts increased as the hydrophobicity of the B_12_ model complexes increased. The hydrophobicity can be varied through chemical modification of the peripheral ester groups placed at the peripheral sites of the corrin skeleton. The HSA microenvironments increased the yield of the acetyl-migrated product compared with the homogenous conditions of the methanol or benzene solutions (Scheme 4). This increase resulted from the effects of suppression of molecular motion and the desolvation of the B_12_ model complex in HSA.

**Scheme 5:**
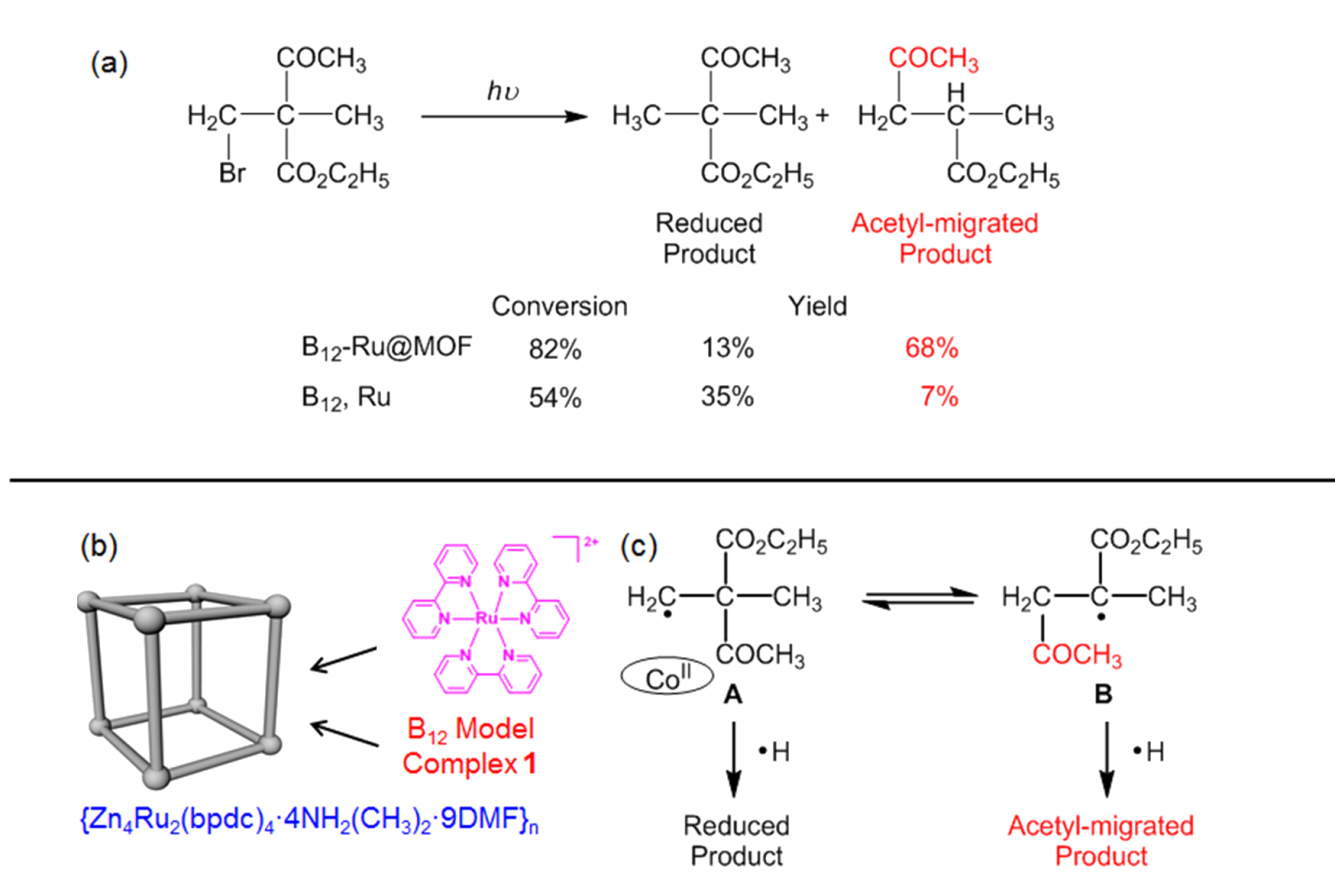
Photochemical carbon-skeleton arrangements mediated by B_12_-Ru@MOF.

MOFs are a class of crystalline materials constructed from metal connecting nodes and molecular building blocks [62–64]. To explore the utilities of the microenvironments provided by MOFs for B_12_ catalytic reactions, a new MOF {Zn_4_Ru_2_(bpdc)_4_·4NH_2_(CH_3_)_2_·9DMF}*_n_* (H_2_bpdc = 4,4′-biphenyldicarboxylic acid) was prepared by the reaction of H_2_bpdc, Ru(bpy)_2_Cl_2_, and a zinc source under solvothermal conditions (bpy = 2,2′-bipyridine, Scheme 5) [41]. The molecular photosensitizer [Ru(bpy)_3_]^2+^ was incorporated into the MOF through adsorption to form Ru@MOF, accompanied by a color change. Furthermore, **1** was effectively immobilized on Ru@MOF, as was confirmed through ESR measurements. The resultant heterogeneous hybrid catalyst B_12_-Ru@MOF successfully mediated the photochemical carbon-skeleton arrangement. Previous studies had demonstrated that the hemolytic cleavage of the Co(III)–C bond of the alkylated complex of **1** generated Co(II) species and an alkyl radical intermediate A [54]. The prolonged lifetime of the radical intermediate A could be provided by the channel of MOF, enabling conversion to the acetyl-migrated radical B. The radicals A and B may abstract hydrogen radicals to form the reduced product and the acetyl-migrated product, respectively. It was noticeable that the catalytic cycle for 1,2-migration was constructed for the B_12_-Ru@MOF system. This stands in contrast to the stoichiometric reactions in the previous B_12_ artificial enzymes. Furthermore, the catalytic process of the B_12_-Ru@MOF system is visible-light-driven through the use of [Ru(bpy)_3_]^2+^ as an alternative to reductases. This serves as a simplified analogy for the B_12_ enzyme-involving system (Figure 2a). The B_12_-Ru@MOF is the best system for the functional simulation of MMCM among our B_12_ artificial enzymatic systems.

### 3. Methyl transfer reactions

**Scheme 6:**
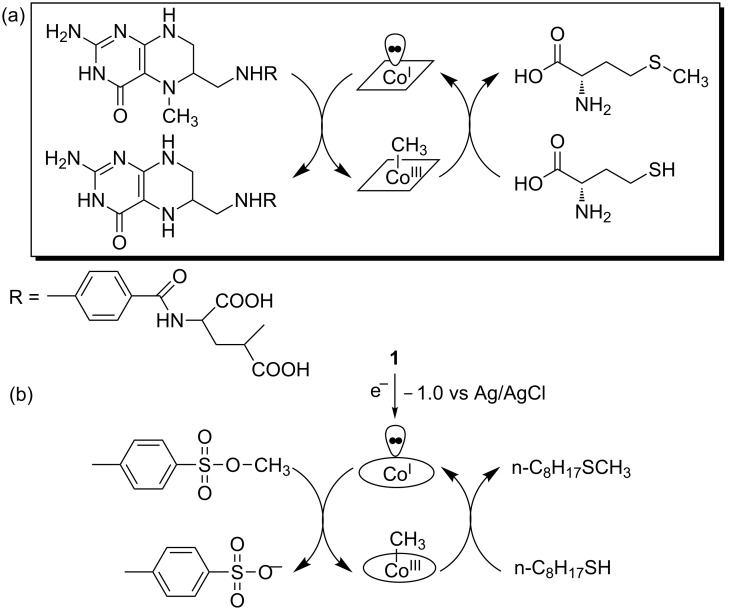
(a) Methyl transfer reaction mediated by B_12_-dependent methionine synthase. (b) Methyl transfer reaction from TsOCH_3_ to 1-octanthiol mediated by **1**.

The B_12_-dependent methionine synthase catalyzes the methyl transfer reaction as shown in Scheme 6a. In the active center of the enzyme, cob(I)alamin accepts the methyl group from methyltetrahydrofolate (CH_3_-H4-folate) and the resultant methylcobalamin donates it to homocysteine [65–66]. Constructing the methyl transfer cycle under non-enzymatic conditions is a challenging issue for chemists. Here, we describe model studies of the methylation of B_12_ derivatives and methyl transfer from methylated B_12_ derivatives. Zn^2+^ ions were considered as the essential cofactors in the enzymatic reactions reported by many researchers [67–69].

#### 3-1. Methyl transfer to thiols

Chemical reductants such as NaBH_4_ or electrochemical reduction could provide Co(I) species, so that α-methylated and β-methylated B_12_ could be formed by the oxidative addition reaction with a methyl donor. The supernucleophile Co(I) species readily react with various methyl halides such as methyl iodide to form a methyl–cobalt complex. Moreover, methanol could also serve as a methyl donor after the activation of the OH group by a Lewis acid such as Zn^2+^ [70–71]. Thiols could also mediate the methylation of **1** with methyl iodide or methyl tosylate (TsOCH_3_) as the methyl donor [72]. Kräutler et al. found an equilibrium methyl transfer between methylcobalamin and the methylated complex of **1** resulting in cob(II)alamin and β-methyl heptamethyl cob(III)yrinate. Such a thermal equilibration takes 16 days at room temperature [73].

Keese et al. successfully constructed a complete methyl transfer cycle from methylamines to 1-hexanethiol as an excellent bioinspired system. The use of Zn and ZnCl_2_ in refluxing ethanol was vital for the bioinspired methyl transfer [74]. Recently, we developed a catalytic methyl transfer system for the first time through electrolysis under non-enzymatic conditions. The methyl transfer from TsOCH_3_ to 1-octanethiol was mediated by controlled-potential electrolysis at −1.0 V vs Ag/AgCl in the presence of **1** at 50 °C (Scheme 6b) [75]. The Zn plate was used as a sacrificial anode and the resultant Zn^2+^ ions was vital for the activation of 1-octanethiol [76]. A similar reaction was successfully mediated by the imine/oxime-type cobalt complex **2a** using zinc powder [77].

#### 3-2. Methyl transfer to inorganic arsenic for the detoxification of arsenic

**Scheme 7:**
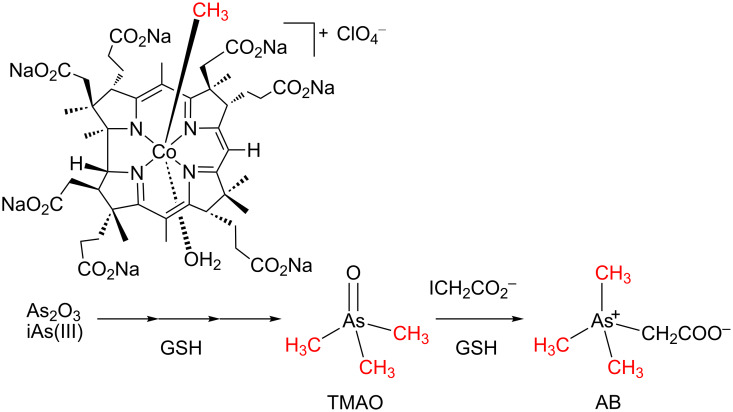
Methyl transfer reaction for the detoxification of inorganic arsenics.

The wide utilization of inorganic arsenics causes large-scale environmental pollution, resulting in very chronic diseases [78]. However, it was known that the toxicity of organic arsenics is generally much lower than inorganic ones. For example, the acute toxicity of arsenobetaine (AB) is about one three-hundredth that of arsenic trioxide [79]; trimethylarsine oxide (TMAO) that is an intermediate in the synthesis of AB also has lower toxicity than inorganic arsenics. Moreover, inorganic arsenics could be converted to methylated arsenics via human or animal metabolism involving a methyltransferase and a reductase [80–82]. Thus, biomimetic transformation from inorganic arsenics to organic arsenics via methyl transfer could be an eco-friendly methodology for the detoxification of arsenic. The B_12_-mimetic methyl transfer reaction for the detoxification of inorganic arsenics has recently been developed. The highly toxic As_2_O_3_ was transformed to AB via TMAO under mild conditions, as shown in Scheme 7 [83–84]. High efficiency transformation of As_2_O_3_ to TMAO was newly achieved with methylated complex of **1** as a methyl donor and GSH as a reductase model.

The methyl transfer reaction to As_2_O_3_ was first examined at 37 °C in Tris–HCl buffer for 24 h. A methylated complex of **1** was proved to be more efficient than the naturally occurring methylcobalamin [84]. More than 95% of As_2_O_3_ was converted into methylarsonic acid (MMA, 67.8%), dimethylarsonic acid (DMA, 27.2%), and TMAO (0.1%) in the reaction of **1**, whereas only 20% conversion of As_2_O_3_ was observed in the reaction of methylcobalamin with lower methylated MMA (17.2%) and DMA (2.8%) as products. When the reaction of the methylated complex of **1** was performed at 100 °C in Tris–HCl buffer for 2 h, As_2_O_3_ was methylated to TMAO with much as 99% yield [83]. Combined with the nearly quantitative conversion of TMAO to AB in the presence of GSH and iodoacetic acid in phosphoric acid–citric acid buffer at 37 °C, a safe and eco-friendly detoxification of inorganic arsenics was developed via methyl transfer reactions mediated by biomimetic vitamin B_12_.

### 4. Dehalogenation reactions

**Scheme 8:**
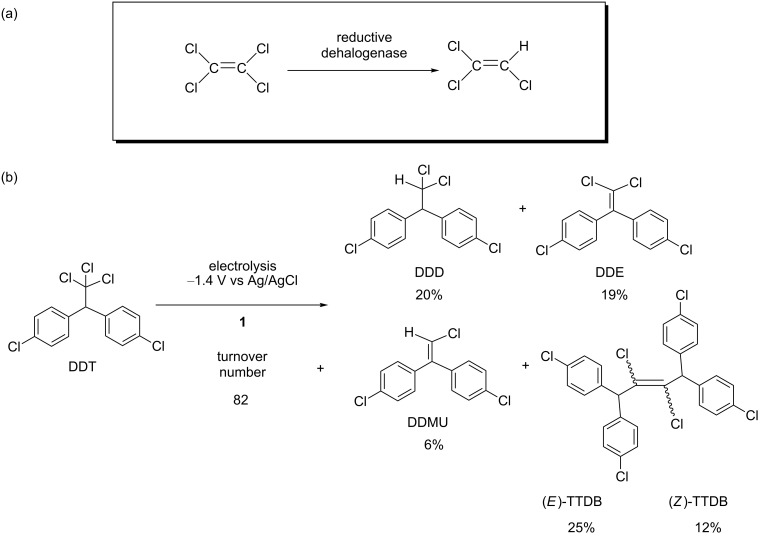
(a) Dechlorination of 1,1,2,2-tetrarchloroethene mediated by a reductive dehalogenase. (b) Electrochemical dechlorination of DDT mediated by **1**.

“Dehalorespiration” is also a model of good catalysts for chemists because the anaerobic metabolism of microbes couples the dehalogenation of organic halides with energy conservation [85]. In some electron transport chains, reductive dehalogenases contain B_12_ derivatives as cofactors [86]. The reductive dehalogenase originating from the anaerobic bacteria, *Sulfurospiririllum multivorans*, uses 1,1,2,2-tetrarchloroethene as a terminal electron acceptor to be reduced to trichloroethene (Scheme 8a) [87]. In electron transport chains, reductases reduce the Co(II) species of the B_12_ cofactor to the Co(I) species in the active site of reductive dehalogenases [88]. The Co(I) species is a key form for electron transfer to a substrate.

#### 4-1. Choice of alternatives to reductases

Although anaerobic microbes can be applied to remediation technologies, the dehalogenation abilities of microbes are equal to the intrinsic abilities of nature in principle. Chemical methods are considered as efficient techniques to directly degrade halogenated pollutants. Completely mimicking the complicated dehalorespiration systems requires tedious efforts. The concept of bioinspired chemistry would be an effective methodology to design sustainable systems. To construct good catalytic dehalogenation systems, the key process is the reduction of Co(II) species of B_12_ derivatives to the Co(I) species in sustainable processes.

Electroorganic synthesis is considered an eco-friendly method for synthetic organic chemistry [89–91]. Clean redox events between electrodes and substrates can be achieved without any chemical redox reagents. The use of mediators enables energy savings with mild applied potentials or small amounts of electricity. We constructed electrochemical catalytic systems for dehalogenation of alkyl halides using **1**. The electron transfer from reductases to B_12_ was replaced with that from the cathodes to B_12_ derivatives [43].

Light-driven organic transformations attract great attention due to their relevance to photosynthesis in nature as an ideal sustainable system [92–94]. In this context, we constructed light-driven catalytic systems using **1** by replacing reductases with semiconductor photosensitizers and molecular photosensitizers. For example, we reported an ultraviolet-light-driven system using titanium dioxide (TiO_2_) semiconductor [95–101]. The conductive band electron of TiO_2_ (*E*_red_ = −0.5 V vs NHE in neutral water) could reduce **1** to form Co(I) species upon irradiation with ultraviolet (UV) light. We also reported a visible-light-driven system with a molecular photosensitizer such as Ru(bpy)_3_^2+^ [39–40,102–103], cyclometalated iridium(III) complexes [104], and organic red dyes [105–107].

#### 4-2. Dechlorination of DDT and related compounds

We developed an electrochemical catalytic system for the dechlorination of 1,1-bis(4-chlorophenyl)-2,2,2-trichloroethane (DDT) that is one of the most problematic persistent organic pollutants (POPs) [108]. The controlled-potential electrolysis of DDT was performed at −1.4 V vs Ag/AgCl in the presence of **1** in DMF/*n-*Bu_4_NClO_4_. The DDT was converted to 1,1-bis(4-chlorophenyl)-2,2-dichloroethane (DDD), 1,1-bis(4-chlorophenyl)-2,2-dichloroethylene (DDE), 1-chloro-2,2-bis(4-chlorophenyl)ethylene (DDMU), and 1,1,4,4-tetrakis(4-chlorophenyl)-2,3-dichloro-2-butene (TTDB, *E*/*Z*) through dechlorination (Scheme 8b) [109]. A turnover number of 82 based on **1** was achieved. Mechanistic investigation revealed that the electrochemically generated Co(I) species of **1** participated in the dechlorination. To recycle the catalyst, ionic liquids are promising solvents due to their excellent electronic conductivity and nonvolatility. Thus, 1-butyl-3-methylimidazolium tetrafluoroborate ([bmim][BF_4_]) was utilized as the solvent in the dechlorination of DDT [110]. During the extraction process, the product and **1** were separated in the organic solvent and ionic liquid layers, respectively. The ionic liquid layer could be recycled for further reactions. More interestingly, the catalytic ability of **1** increased nearly four times the reaction using DMF as solvent. This was consistent with the Hughes–Ingold prediction of solvent polarity effects on reaction rates [111].

**Scheme 9:**
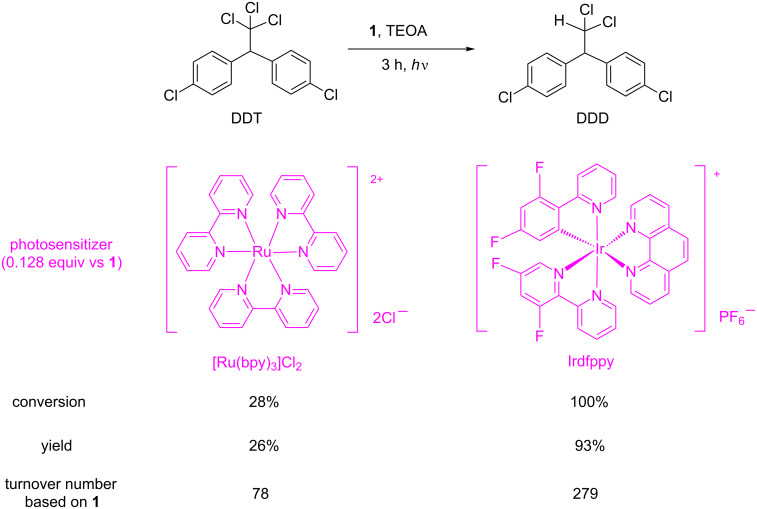
Visible-light-driven dechlorination of DDT using **1** in the presence of photosensitizers.

We also developed a visible-light-driven catalytic system for the dechlorination of DDT using **1** as catalyst and [Ru(bpy)_3_]Cl_2_ as photosensitizer [102]. The redox potential of [Ru(bpy)_3_]Cl_2_ for Ru(II)/Ru(I) couple is −1.35 V vs SCE in CH_3_CN. Thus, **1** was reduced to the Co(I) species by the photosensitizer in the presence of triethanolamine (TEOA) as sacrificial reductant on irradiation with a 500 W tungsten lamp in ethanol. DDT was successfully converted to DDD, DDE, and TTDB (*E*/*Z*). The recycled use of **1** and [Ru(bpy)_3_]Cl_2_ was also achieved using an ionic liquid as the reaction medium [103]. Recently, we have found that cyclometalated iridium(III) complexes such as Irdfppy [112] are superior to [Ru(bpy)_3_]Cl_2_ in terms of their photosensitization abilities in visible-light-driven B_12_ catalytic systems (Scheme 9) [104]. This was probably due to the gradual decomposition of [Ru(bpy)_3_]Cl_2_ under visible light irradiation. This is consistent with the report by Yoon et al. in which light irradiation to Ru(bpy)_3_^2+^ resulted in rapid decomposition during the photocatalytic reaction [113]. It was remarkable that a significantly high turnover number based on **1** (10,880) was obtained in the prolonged reaction with Irdfppy. Quenching experiments with time-resolved photoluminescence spectroscopy revealed that the oxidative quenching of the excited state of Irdfppy favorably proceeds over the reductive quenching mechanism. The combination of **1** and Irdfppy offers the best choice for the dechlorination of DDT among our light-driven systems in terms of both catalytic activity and visible-light harvesting.

In relation to the reactivity of **1** with DDT, interesting reactions of trichlorinated organic compounds have recently been investigated [100,114]. The B_12_-TiO_2_ hybrid catalyst converted trichlorinated organic compounds into esters and amides by UV light irradiation in the presence of oxygen, whereas dichlorostilbenes (*E* and *Z* forms) were formed under nitrogen atmosphere from benzotrichloride [100]. It was noticeable that an oxygen switch in dechlorination was successfully demonstrated. A benzoyl chloride was identified as an intermediate of the esters and amides. The aerobic electrolysis of trichlorinated organic compounds was also mediated by **1** to yield esters and amides [114]. These reactions are important in terms of fine chemical production from trichlorinated organic compounds through easy operations (i.e., in air at room temperature).

### 5. Radical-involved organic synthesis

B_12_ derivatives can mediate various molecular transformations in addition to the above three-type catalytic reactions. In particular, alkylated complexes can generate radicals through the cleavage of the Co(III)–C bonds upon light irradiation, heating, or electrochemical reduction. In addition, the corrin-ring of the B_12_ derivatives is tolerant to free radicals, as described above. Thus, alkylated complexes have been used for radical-mediated organic synthesis such as halide coupling, alkene coupling, and addition to double bonds [7,26–27]. In particular, the Co(III) form of **1** has recently been found to catalyze atom transfer radical addition of alkyl halides to olefins (phenyl vinyl sulfone and acrylates) in the presence of NaBH_4_ [115]. In addition, a new light-driven method for generating acyl radicals from 2-*S*-pyridyl thioesters was developed through the use of vitamin B_12_ [116]. Furthermore, cobalester, an amphiphilic vitamin B_12_ derivative with six ester groups and a nucleotide loop, has recently been developed to show good catalytic activity for C–C bond forming reactions [117–118].

**Scheme 10:**
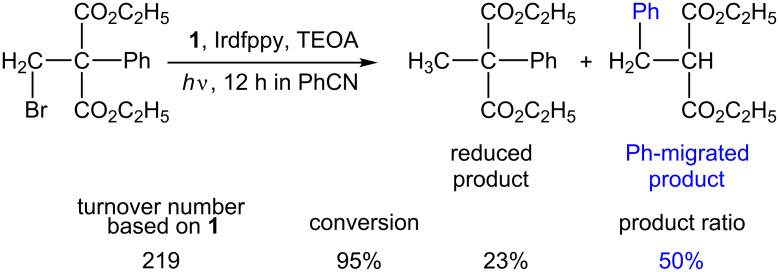
1,2-Migration of a phenyl group mediated by the visible-light-driven catalytic system composed of **1** and Irdfppy.

The above-mentioned visible-light-driven system composed of **1**, and Irdfppy system was used for radical-mediated isomerization reactions. Visible-light irradiation of diethyl 2-bromomethyl-2-phenylmalonate produced the phenyl-migrated product (Scheme 10) [104]. The product distribution highly depended on the solvents. The yield of phenyl-migrated products relative to those of simple reduced products significantly increased in PhCN, a poor hydrogen radical donor solvent, compared with those in EtOH and CH_3_CN. Similar phenyl migration was achieved in the UV-light-driven system of the B_12_-TiO_2_ hybrid catalyst [96–98]. The involvement of a radical species was confirmed by the spin-trapping technique followed by the ESR measurements.

**Scheme 11:**
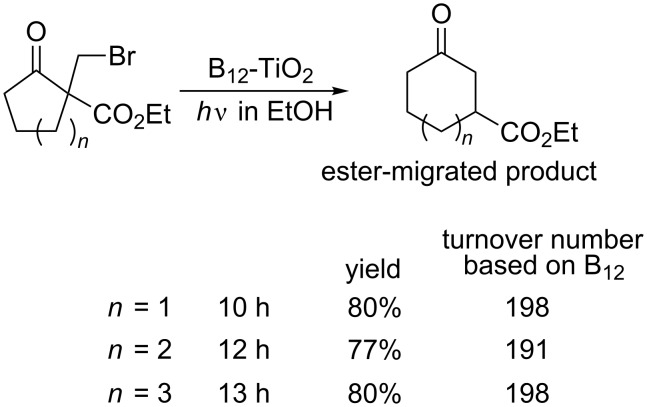
Ring-expansion reactions mediated by the B_12_-TiO_2_ hybrid catalyst with UV-light irradiation.

The B_12_-TiO_2_ hybrid catalyst also mediated the ring-expansion reactions of alicyclic ketones with carboxylic ester and bromomethyl groups (Scheme 11) [96,98]. The products involving six-, seven-, and eight-membered rings were obtained through isomerization with 1,2-migration of the ester groups. The B_12_-TiO_2_ hybrid catalyst can be regarded as a good alternative for conventional radical-involved organic syntheses using tin compounds.

**Scheme 12:**
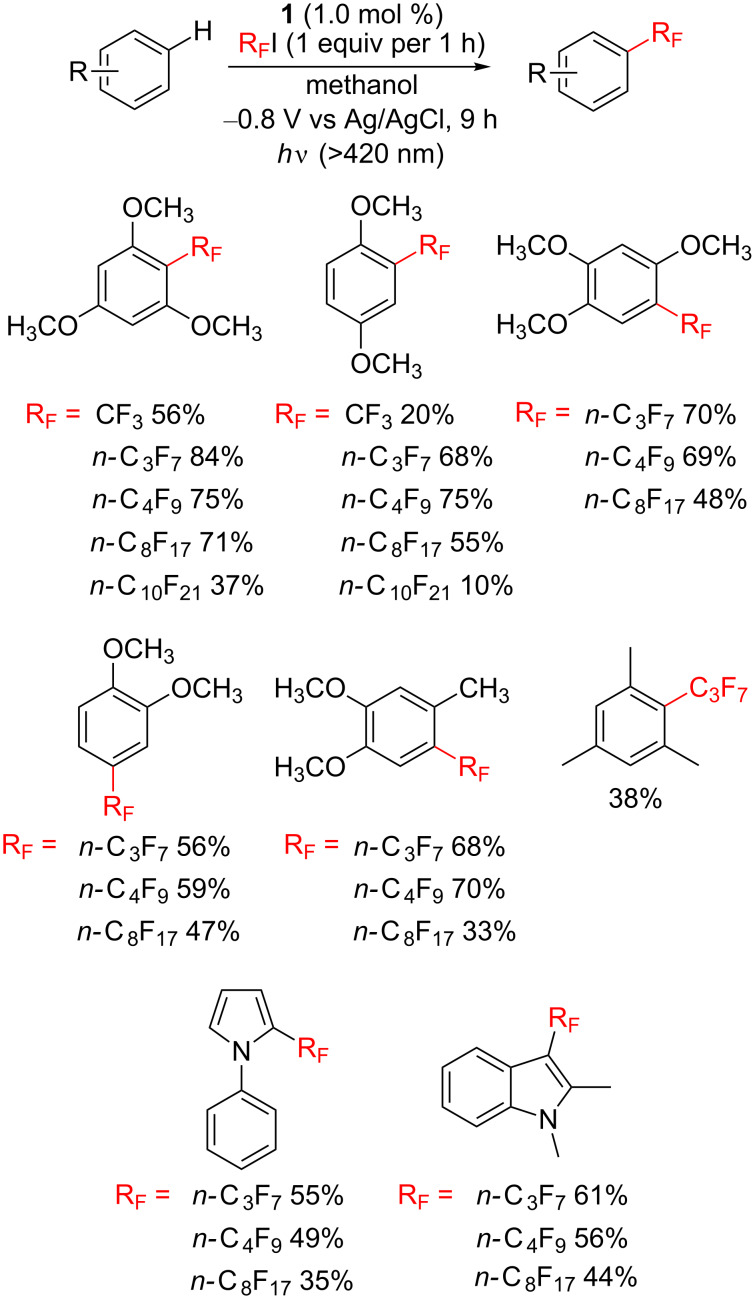
Trifluoromethylation and perfluoroalkylation of aromatic compounds achieved through electrolysis with catalyst **1**.

Recently, we discovered that the B_12_ derivative **1** can mediate trifluoromethylation and perfluoroalkylation of aromatic and heteoaromatic compounds by means of electrolysis [119–120]. Introducing trifluoromethyl and perfluoroalkyl groups (R_F_) into organic compounds is an important target in organic synthesis because the corresponding fluoroalkylated molecules have received significant interest because of their metabolic stability and superior electron-withdrawing and lipophilic properties [121]. The controlled-potential electrolysis of cost-effective fluoroalkylating reagents with carbon–iodine bonds R_F_I (R_F_ = CF_3_, *n*-C_3_F_7_, *n*-C_4_F_9_, *n*-C_8_F_17_, and *n*-C_10_F_21_) was carried out at −0.80 V vs Ag/AgCl in the presence of **1** in methanol/*n*-Bu_4_NClO_4_ to form Co(III)–R_F_ complexes with deiodination. These complexes released R_F_ radicals on the Co(III)-bond cleavage through visible-light irradiation. The resultant radicals reacted with aromatic reagents to form the target products through direct C–H functionalization (Scheme 12).

## Conclusion

In this review, we described biomimetic and bioinspired catalytic reactions with B_12_ enzyme functions, with a classification into the corresponding three enzyme subfamilies. A variety of B_12_ enzymes mediate various molecular transformations, in conjunction with other enzymes. Bound apoenzymes maximize the potential ability of B_12_ as a molecular catalyst. We conceptually broke up natural systems involving B_12_ enzymes into pieces and artificially assembled them again in a unique fashion. The resultant biomimetic and bioinspired systems provide new insights into designing catalytic systems in terms of green and eco-friendly reactions.
